# Supplementary figures and images for: Complete plastid genome of Iris orchioides and comparative analysis with 19 Iris plastomes
Source: PLoS One. 2024 Apr 5;19(4):e0301346. doi: 10.1371/journal.pone.0301346 (PMC10997070; doi:10.1371/journal.pone.0301346)

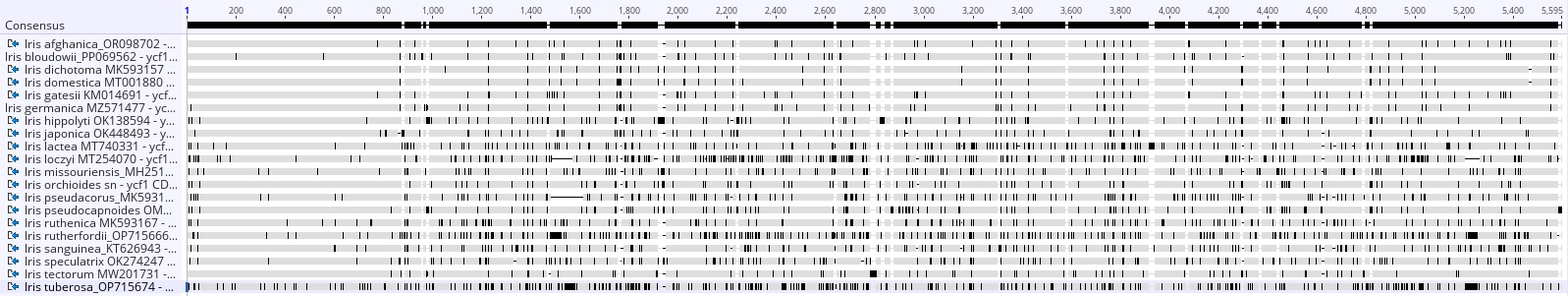

Supplement: S1 Fig — (JPG) [file pone.0301346.s001.jpg]

## matK

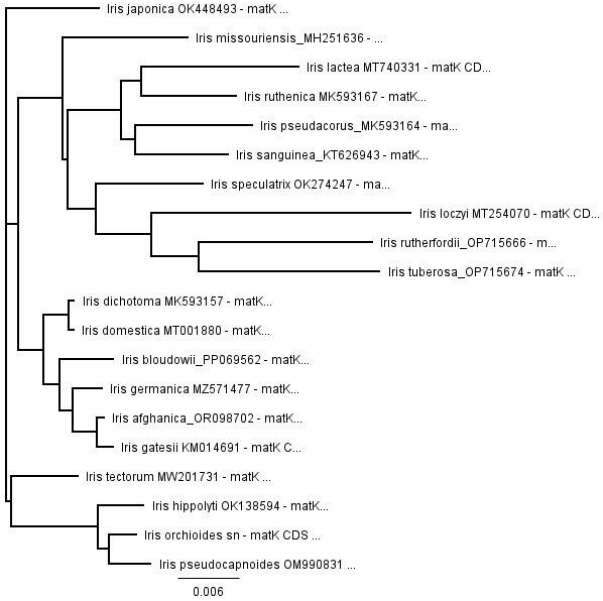

## ndhF

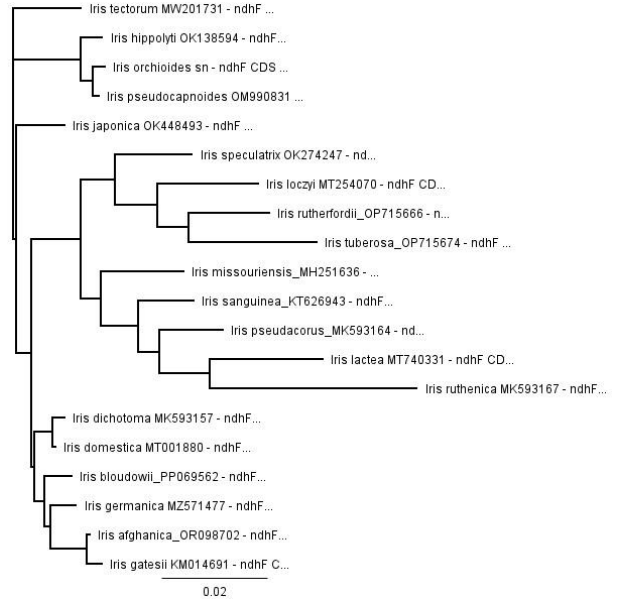

## rpoC2

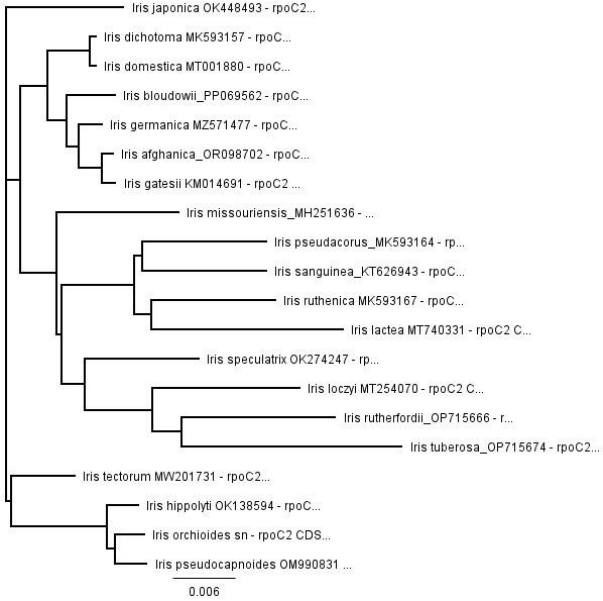

## ycf1

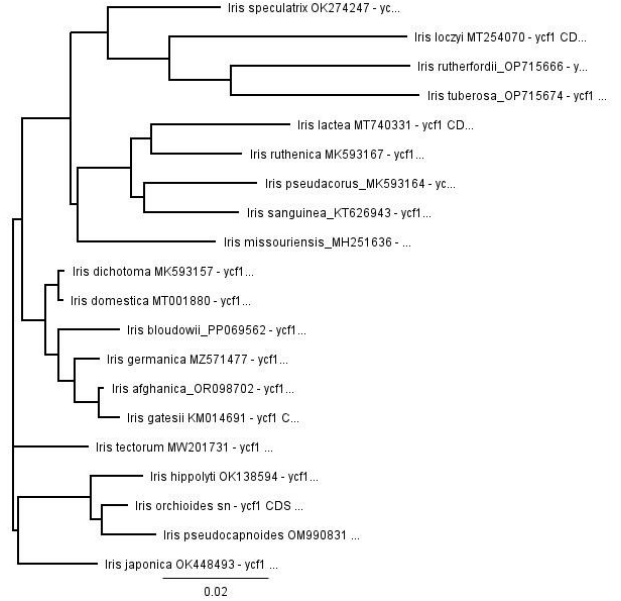

## ycf2

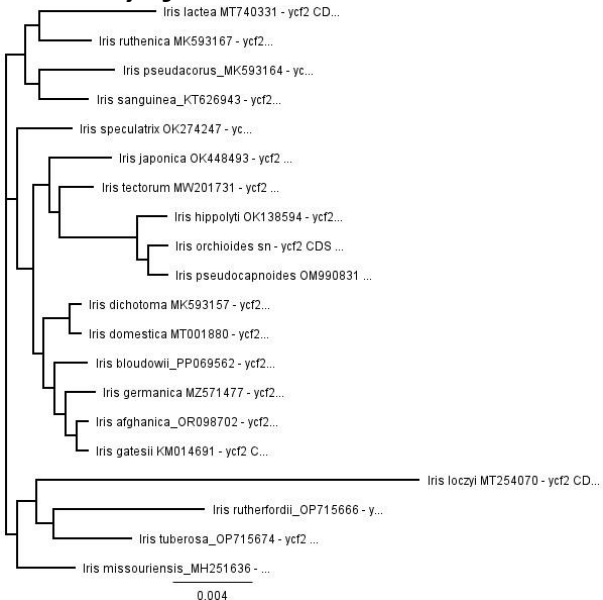

*ndhG-ndhI*

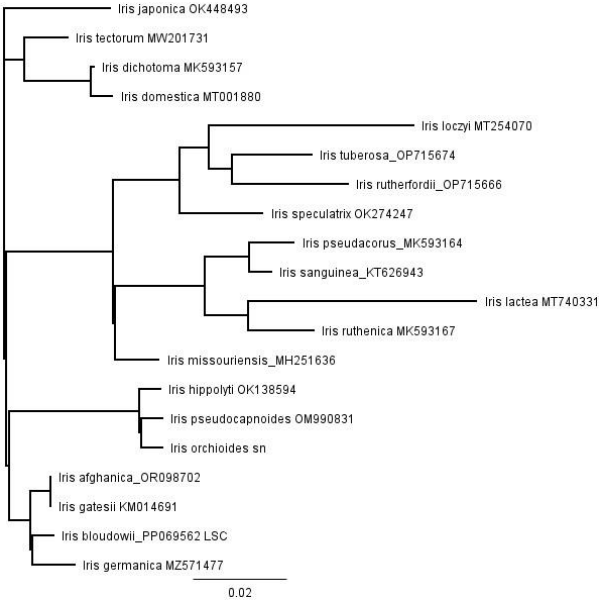

*petA-psbJ*

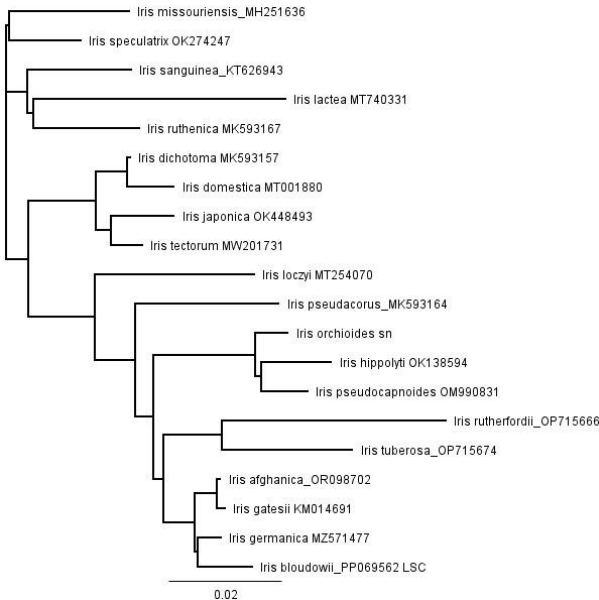

*psbK-trnQ*

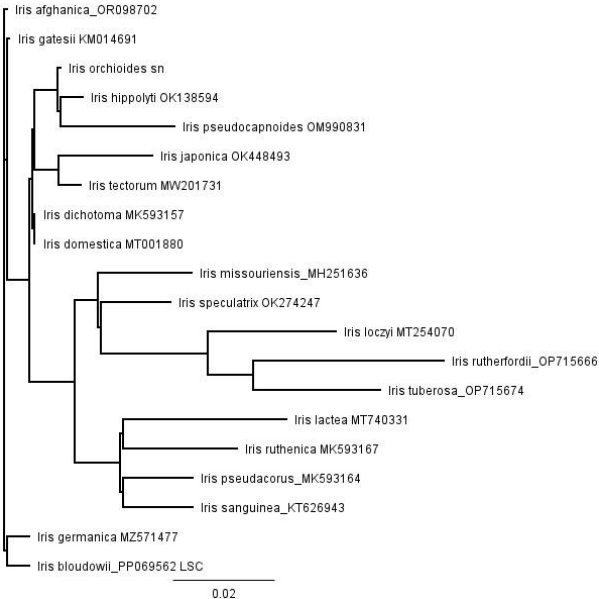

*rpoB-trnC*

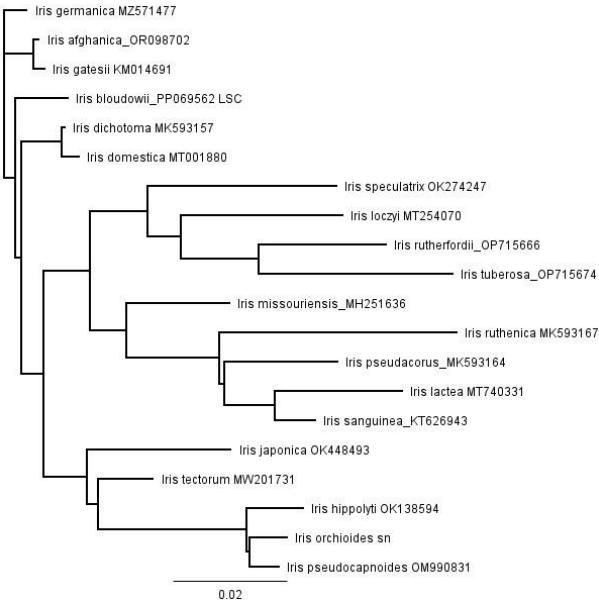

*ycf1-rps15*

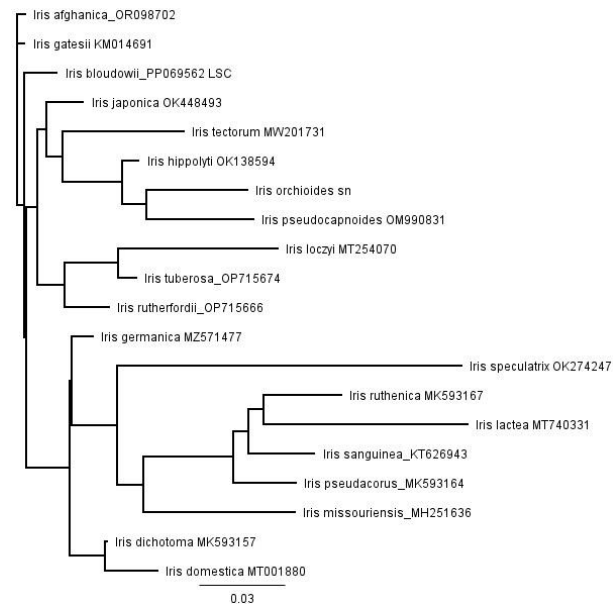

Supplement: S2 Fig — (PDF) [file pone.0301346.s002.pdf]
