# Supplementary material for: Complete plastid genome of Iris orchioides and comparative analysis with 19 Iris plastomes
Source: PLoS One. 2024 Apr 5;19(4):e0301346. doi: 10.1371/journal.pone.0301346 (PMC10997070; doi:10.1371/journal.pone.0301346)
Supplement: Attachment — Submitted filename: Response to reviewers_PLOS_RV_1 (1).docx [file pone.0301346.s008.docx]

Ref: Submission ID PONE-D-23-41497

**Responses to the reviewers**

We appreciate the insightful comments from anonymous reviewers and the handling editor. The manuscript has been improved based on the comments. To navigate the updates in the revised manuscript, please use the review function in MS word. We made the changes available using the track and change function in MS word.

**Editor comments**
Both reviewers questioned the reason for selecting 13 additional plastid genomes among the 40 reported within the genus Iris. Please provide theoretical background for this decision. Additionally, parameters for the selection of 5 genes for further use in phylogenetic analyses in the genus Iris are not clearly presented. Some disagreements have also been noted by both reviewers between the M&M and the Results sections. The authors should put additional effort to clarify these inconsistences and to properly discuss them in the Discussion section.

**Response:** Thank you for the valuable comments. We understand that 13 Iris plastomes seems very limited number considering the currently available data (~90 Iris plastomes). When we analyzed the data, there were only ~40 accessions available. We indeed used all available Iris plastome data for phylogenetic inference. However, for the comparative analysis, we tried to simplify the comparison because there is no point in comparing all accessions as plastomes are very conserved among taxa in Iris. Additionally, the figures depicting the results would likely be hard to read with 40 or more taxa included, and thus blur the messages of the plastome comparison. Therefore, we only selected one or couple species from each subgenus for the comparative analysis. The ms preparation and submission process took quite some time and now over 80 Iris plastome data are available. We think that adding more plastome data to compare the plastomes at section level now is possible. Therefore, we added more data and redo the whole analyses. In the revised manuscript, we compared ~20 taxa by adding 7 more Iris plastomes which will cover at least one or more taxa from each section. The rationale to include 20 plastomes out of 84 were included in the revised manuscript. We also inferred the phylogeny of 84 Iris accession available.

Please use the term "plastid" instead of "chloroplast" and "CP" throughout the text (including the main title), because various plastid forms (chloroplasts, chromoplasts, leucoplasts, amyloplasts, and elaioplasts) contain the same genome as chloroplasts and from the section "Sampling, DNA isolation, and sequencing", I see the authors did not isolated chloroplasts from the other plastids prior to DNA extraction.

**Response:** Thank you for the comments. We corrected the term error throughout the ms.

Abstract: I could not understand the statement "genomic information for the Iris chloroplast is limited". Can you please rephrase it to clarify its meaning. In the next sentence, the phrase "of the Iris chloroplast" implies that all Iris species contain the same chloroplast. Would "of plastids within the genus Iris" be better? In the same sentence, please shorten the genus name in "Iris orchioides" as "I. orchioides".

**Response:** Thank you for the comments. We rewrote the problematic sentence and corrected errors as suggested.

Further in Abstract: what "five selected sites" is related to? Where are these sites? Can you be more precise? In the next sentence, "five identified genes" does not tell much. First, how were they identified? Maybe selected? And, of course, please list the selected genes and criteriums for their selection here.

**Response:** Thank you for the comments. We reorganized the part in the abstract to clearify in the revised ms.

Introduction: Please italicize "Juno", as it presents a synonym for the genus.

**Response:** Thank you for the comments. We corrected the error.

**Reviewer Comments:**
**Reviewer 1**
Reviewer #1: The researchers successfully assembled the chloroplast genome of Iris orchioides and conducted comparative genomic analysis with the chloroplast genomes of 13 previously published Iris species. They identified highly polymorphic gene regions within the Iris chloroplast genome, utilizing these as markers to identify the phylogeny of Iris. The data analysis appears to be robust, lending substantial support to the majority of the conclusions drawn in this study. However, the study still presents several issues.

The key issues identified in the study are:
1. The paper states that 40 chloroplast genome sequences of Iris species are available. However, the reason for selecting 13 of these for comparative analysis with the I. orchioides chloroplast genome sequence is not clear. The text should explicitly justify this selection.

**Response:** Thank you for the valuable comments. We understand that 13 Iris plastomes seems very limited number considering the currently available data (~90 Iris plastomes). When we analyzed the data, there were only ~40 accessions available. We indeed used all available Iris plastome data for phylogenetic inference. However, for the comparative analysis, we tried to simplify the comparison because there is no point in comparing all 40 accessions as plastomes are very conserved among taxa in Iris. Additionally, the figures depicting the results would likely be hard to read with 40 or more taxa included, and thus blur the messages of the plastome comparison. Therefore, we only selected one or couple species from each subgenus for the comparative analysis. The ms preparation and submission process took quite some time and now over 90 Iris plastome data are available. We think that adding more plastome data to compare the plastomes at section level now is possible. Therefore, we added more data and redid the whole analyses. In the revised manuscript, we compared ~20 taxa by adding 7 more Iris plastomes which will cover at least one or more taxa from each section. The rationale to include 20 plastomes out of 84 were included in the revised manuscript (method section). We also inferred the phylogeny of all 84 Iris accessions available.

2. The interpretation of the constructed phylogenetic tree is missing, as is a discussion on how these results contribute to differentiating Iris species. Furthermore, there is no comparison of these findings with prior phylogenetic analyses based on morphology and nuclear genes.

**Response:** Thank you for the comments. We added the interpretation of the inferred tree and compared the tree with formerly published data in the revised ms as suggested.

3. The absence of population-level chloroplast genome data precludes inferring the suitability of the identified markers for population data.

**Response:** Thank you for the comments. We agree that population level plastid genome data would be great to add if enough grant to sequence hundreds of samples was given. However, our grant was too limited to sequence such many samples. In addition, our focus for this research was not particularly to determine population level genetic variation within an *Iris* species. As presented in the introduction, our primary objectives are to characterize the architecture of Iris plastid genomes and identify good plastid markers for phylogenetic analyses. We probably weren’t very clear about this in some section of the discussion. To clarify that our research is not fully focused on population level studies, we revised and added a couple sentences in the discussion section.

4. The methodology for narrowing down the ten regions with high Pi values to five candidate sites remains unclear. Detailed explanation in the text is required.

**Response:** Thank you for the comments. First to be clear, the best alternative to the whole plastome phylogeny was the one inferred from the concatenated data of the five high PI sites. We clarified that in the main text. As the reviewer suggested, we also added how we determined the candidate site in the methods section.

5. The conclusion drawn from Figure 7, suggesting that the ten genes approximated the entire chloroplast genome in inferring the phylogeny of Iris, requires further clarification and evidence.
**Response:** Thank you for the comments. We are sorry for the confusion. Fig. 7 showed the congruence between the plastome phylogeny and the phylogeny with the selected data which is the concatenated data of three high PI genes. The evidence is the highest TreeDist score and similarity in overall tree topology. In the revised ms, we clarified the justification by providing TreeDist score and clear explanation.

The minor issues identified in the study are:
1. Numerous textual errors are present notably in the method section (specific line numbers not provided) and line 182.

**Response:** Thank you for the comments. We edited the entire method section in the revised ms as suggested.

2. Figure 1 lacks an indication of the transcription direction.

**Response:** Thank you for the comments. We added the transcript direction in fig. 1 as suggested.

3. The font of Table 1 should be modified.

**Response:** Thank you for the comments. We reformatted the table 1 as suggested.

4. The identification of outgroups in Figures 5 and 6 is unclear, and the significance of the various colors used is not delineated.

**Response:** Thank you for the comments. We reorganized the figures and revised legends as suggested.

5. In Figure 2, areas of high polymorphism should be emphasized to enhance visibility for readers.

**Response:** Thank you for the comments. We highlighted the high polymorphic regions in the figure as suggested.

6. The manuscript’s English language quality requires substantial improvement.

**Response:** Thank you so much for the valuable comments. All authors edited the entire ms to improve the language quality.

**Reviewer 2**
Reviewer #2: In this study, the authors newly sequenced the cp genome of Iris orchioides and comparatively analyzed the cp genomes of other Iris species. This study had a scientific significance. However, some questions need to be revised as follows:

1. In the introduction section, S, and some groups fail to form monophyletic groups in most molecular phylogenies?? The phylogenetic relationships were not solved using some molecular markers? Please clarify it in detail. The classification of genus Iris should be detailed, and the information of subgenus was mentioned in the main text.

**Response:** Thank you for the comments. The sentence was mistakenly written. We clarified the text and edited the errors. We also re-analyzed phylogenetic section with more samples added as requested by another reviewer. Therefore, the results and discussion for the Iris phylogeny was largely revised.

2. The cp genomes of 40 Iris species were released. Why only 13 species were used to compare in this study?

**Response:** Thank you for the valuable comments. We understand that 13 Iris plastomes seems very limited number considering the currently available data (~90 Iris plastomes). When we analyzed the data, there were only ~40 accessions available. We indeed used all available Iris plastome data for phylogenetic inference. However, for the comparative analysis, we tried to simplify the comparison because there is no point in comparing all 40 accessions as plastomes are very conserved among taxa in Iris. Additionally, the figures depicting the results would likely be hard to read with 40 or more taxa included, and thus blur the messages of the plastome comparison. Therefore, we only selected one or couple species from each subgenus for the comparative analysis. The ms preparation and submission process took quite some time and now 84 Iris plastome data are available. We think that adding more plastome data to compare the plastomes at section level now is possible. Therefore, we added more data and redid the whole analyses. In the revised manuscript, we compared ~20 taxa by adding 7 more Iris plastomes which will cover at least one or more taxa from each section. The rationale to include 20 plastomes out of 84 were included in the revised manuscript. We also inferred the phylogeny of 84 Iris accession available.

3. In the method section, the results of phylogenetic analysis were not consistent with the description of the method.

**Response:** Thank you for the comments. As mentioned above, we greatly improved the phylogenetic analysis and the results section. In the revised ms, the methods and results are consistent.

4. In the table 1, the classification of each species had better to be added.

**Response:** Thank you for the valuable comments. We added the taxonomic position of the taxa included in the analysis as suggested.

5. In the results section, the description of the figure 6 and 7 was not consistent with the results. In addition, figure 5 showed the phylogenetic tree based on the cp genome data. However, another tree had also included this tree. Figure 5 should be deleted.

**Response:** Thank you for pointing it out. We corrected the error in the revised ms.

6. The author selected ten regions with high-pi values, their phylogenetic tree should be also shown in the text.

**Response:** Thank you for the comments. As mentioned above, we added more data and redid the PI and phylogenetic analysis. The selected data were slightly changed with the reanalysis. Regardless, we added the ML trees with the top 10 high PI regions in the supplementary information.

7. What method was used to select the five regions? Whether was the five markers suit for these analyzed species? How about other species?

**Response:** Thank you for the comments. First to be clear, the best alternative to the whole plastome phylogeny was the one inferred from the concatenated data of the five high PI sites not each of the 5 sites. We are sorry for the confusion. That part was also clarified in the main text. We also added how we determined the candidate site in the methods section.

8. Where was the Table S5?

**Response:** Thank you for the comment. The table is inserted in the supplementary information file.

9. The title of axis should be added in figure 7.

**Response:** Thank you for the comment. We added the axis title in the figure.
